# Cellular glutathione content in the organ of Corti and its role during ototoxicity

**DOI:** 10.3389/fncel.2015.00143

**Published:** 2015-04-28

**Authors:** Paromita Majumder, Michael R. Duchen, Jonathan E. Gale

**Affiliations:** ^1^UCL Ear Institute, University College LondonLondon, UK; ^2^Department of Cell and Developmental Biology, University College LondonLondon, UK

**Keywords:** antioxidant, aging, ototoxicity, aminoglycosides, hair cells, organ of Corti, supporting cells, inner ear

## Abstract

Glutathione (GSH) is the major scavenger of reactive oxygen species (ROS) inside cells. We used live confocal imaging in order to clarify the role of GSH in the biology of the organ of Corti, the sensory epithelium of the cochlea, before, during and after the onset of hearing and in ~1 year old mice. GSH content was measured using monochlorobimane (MCB), a non-fluorescent cell permeant bimane that reacts with GSH, forming a fluorescent adduct through a reaction catalyzed by glutathione-S-transferase. GSH content increased significantly in inner hair cells during maturation in young adult animals, whereas there was no significant change in the outer hair cells. However, the GSH content in inner hair cells was significantly reduced in ~1 year old mice. The GSH content of supporting cells was comparatively stable over these ages. To test whether the GSH content played a significant protective role during ototoxicity, GSH synthesis was inhibited by buthionine sulfoximine (BSO) in organotypic cochlear explant cultures from immature mice. BSO treatment alone, which reduced GSH by 65 and 85% in inner hair cells and outer hair cells respectively, did not cause any significant cell death. Surprisingly, GSH depletion had no significant effect on hair cell survival even during exposure to the ototoxic aminoglycoside neomycin. These data suggest that the involvement of ROS during aminoglycoside-induced hair cell death is less clear than previously thought and requires further investigation.

## Introduction

The tri-peptide gamma-glutamyl-cysteinyl-glycine (glutathione) is the major cellular antioxidant to combat reactive oxygen species (ROS). There are three major reservoirs of glutathione inside eukaryotic cells: ~90% in cytosol, ~10% in the mitochondria, and <1% in the endoplasmic reticulum. In cytoplasm, under normal physiological conditions, the ratio of reduced glutathione (GSH) to the oxidized disulphide form (GSSG) varies from 10:1 to 100:1 (Hwang et al., 1992; Lu, 1999). GSH is an antioxidant and a substrate for anti-oxidant enzymes such as selenium-dependent GSH peroxidase that neutralizes H_2_O_2_ and other free radicals. GSH is converted back to GSSG during this activity. A cellular redox cycle is completed when GSSG is recycled back to its reduced form by GSSG-reductase at expense of NAPDH.

ROS are a natural side product of oxidative phosphorylation, being produced at many positions along the electron transport chain in the mitochondria (Gutteridge and Halliwell, 2000; Balaban et al., 2005). ROS molecules react with DNA, proteins and, lipids causing mutations and DNA–strand breaks, protein oxidation and, lipid peroxidation. A major contributor to the oxidative damage inside the cell is H_2_O_2_, which can be converted to superoxide (•O^−^_2_) and the hydroxyl radical (•OH).

In the inner ear, oxidative stress has long been associated with cellular damage that results from extracellular insults such as noise-induced hearing loss, aging and ototoxic drugs e.g. the aminoglycoside antibiotics and the cancer therapeutics, carboplatin and cisplatin (Yamane et al., 1995; Clerici et al., 1996). There is surprisingly little direct evidence that ROS formation is causal in cases of acquired hearing loss. Support for the ROS theory for hair cell loss comes from experiments in which antioxidants are found to ameliorate the effects of aminoglycoside ototoxicity and noise-induced hearing loss (Pierson and Gray, 1982; Hoffman et al., 1987; Quirk et al., 1994; Lautermann et al., 1995, 1997; Dehne et al., 2000). A number of mouse models also support the theory that ROS contribute significantly to acquired hearing loss including a targeted mutation of glutathione peroxidase-1 and a superoxide dismutase knockout (McFadden et al., 1999a,b; Ohlemiller et al., 2000). Nutrient deprivation, which is presumed to deplete the supply of GSH in hair cells, has been shown to enhance cell death during aminoglycoside ototoxicity, however as the authors indicated those results were correlative rather than direct (Lautermann et al., 1995). During aminoglycoside ototoxicity it has been proposed that aminoglycosides interact with transition metals inside the hair cell generating ROS and hydroxyl radicals due to the Fenton reaction (Priuska and Schacht, 1995) and the free radicals go on to trigger cell death pathways resulting in the apoptosis, see (Forge and Schacht, 2000).

An aim of this work was to evaluate the distribution of one of the primary sources of ROS buffering, GSH, in the organ of Corti, to assess the relative vulnerability of different cell types to oxidative damage. GSH content was measured in the organ of Corti at different ages through maturation of the organ into adulthood. In addition we assessed GSH content in aged (~1 year old) mice. We used live imaging in *ex vivo* auditory bullae preparations to quantify the fluorescent product of a reaction between monochlorobimane (MCB) and endogenous GSH. This allowed direct assessment of identifiable cells in a native, accessible environment in which the normal cell-cell interactions are maintained. In order to explore the role of GSH during aminoglycoside toxicity, we exposed organotypic cochlear explant cultures to the gamma-glutamylcysteine synthetase inhibitor buthionine sulfoximine (BSO) to deplete cellular GSH (Meister, 1983; Drew and Miners, 1984), then challenged with neomycin and evaluated hair cell survival.

## Materials and methods

### Postnatal and adult cochlea isolation and culture

The animals were sacrificed in accordance with the United Kingdom Animals (Scientific Procedures) Act of 1986. Auditory bullae were isolated from C57BL/6 mice at postnatal days 4–6 (P4–P6), at hearing onset (P14–P16) and in both young (P33–P47) and aged (P357–P424, ~1 year old) adults. Auditory bullae were removed and transferred into Leibovitz's L15 (L15; LifeTech, UK) and then a window opened in the apical turn (Mammano and Ashmore, 1993); Reissner's membrane was peeled away and the stria vascularis removed in order to gain visual access to the organ of Corti. All acute experiments were performed in L15 solution.

For the aminoglycoside and BSO experiments that required longer incubations, cochleae from P4 to P6 mice were dissected out and placed in explant culture. Auditory bullae were removed and transferred into medium 199 (M199H, LifeTech, UK) supplemented with penicillin (10 U/ml) and fungizone (25 ng/ml). The cochleae were isolated from the surrounding cartilagenous capsule, Reissner's membrane was cut and removed but stria vascularis was maintained. The cochlea was cut into three and the apical, middle and basal turns then placed onto Cell-Tak® cell and tissue adhesive (BD Biosciences, UK)-coated Matek® (Ashland, MA, USA) culture dishes. Cell-Tak® was diluted in 0.1M NaHCO_3_ to a final concentration of 70 μg/ml. Cochlear explants were subsequently incubated in DMEM/F-12 (LifeTech, UK) supplemented with 1% fetal bovine serum (FBS, LifeTech, UK) and maintained at 37°C in a 5% CO_2_/95% air atmosphere.

During aminoglycoside exposure experiments, cochlear explant cultures were maintained in HEPES-buffered Hanks Balanced salt solution (10 mM HEPES) adjusted to pH 7.3 with NaOH.

### Glutathione staining and imaging

Monochlorobimane (MCB) is a non-fluorescent bimane which is freely permeable across the cell membrane and forms a fluorescent adduct when combined with reduced glutathione (GSH) in a reaction catalyzed by glutathione s-transferase. MCB (Sigma-Aldrich, UK) was prepared as a 50 mM stock solution in DMSO and used at a final concentration of 50 μM. Our initial experiments confirmed that this reaction reached steady-state within 40 min (Supplementary Figure 1). Therefore, in all experiments MCB was applied for 40 min prior to imaging for cellular GSH measurement (Keelan et al., 2001). The conjugated GSH-MCB was imaged using multiphoton excitation from a Chameleon-XR Ti:Sapphire laser (Coherent, UK) tuned to 780 nm and fluorescence emission was captured using a 435–485 nm bandpass filter. The Zeiss 510NLO Axioskop and all other hardware was controlled by Zeiss LSM software. Image stacks were acquired at 2 μm intervals using a 40 × (NA 0.8) water immersion objective. All experiments were performed at room temperature (20–23°C) keeping all confocal imaging parameters constant between experiment e.g., laser power, pinhole, detector, sensitivity.

### Neomycin and BSO treatment, tissue fixation, and immunostaining

In order to reduce cellular GSH, cochlear explants were treated with 200 μM buthionine sulfoximine (BSO, Sigma-Aldrich, UK) for 16–18 h. BSO is an irreversible inhibitor of gamma-glutamylcysteine synthetase (Drew and Miners, 1984; Sun et al., 2005). At the end of the BSO treatment the explants were loaded with MCB and MCB-GSH fluorescence data were collected as described above. In separate ototoxicity experiments, BSO pre-treated and untreated control explants were incubated with 1 mM neomycin (Sigma-Aldrich, UK) for 3 or 6 h at 23–25°C, prior to fixation with 4% paraformaldehyde in 0.1 M phosphate buffer solution (PBS, pH 7.2) for 30–40 min. The tissue was rinsed three times with PBS and then incubated in blocking solution (PBS/0.5% TritonX-100 with 10% secondary host antibody serum) for 2 h, prior to incubation with either a polyclonal or monoclonal anti-myosin VIIA primary antibody (1:1000, Proteus and 1:250 Developmental Studies Hybridoma Bank, Iowa respectively) in blocking solution overnight at 4°C. Samples were then washed in PBS and incubated for 2 h at room temperature with 4′,6′-diamidino-2-phenylindole (DAPI 1 μM), Alexa Fluor 647 phalloidin (33 nM) and goat anti-rabbit-Atto488 or goat anti-mouse-Atto488 secondary antibodies in blocking solution. After labeling, the explants were rinsed three times with PBS and imaged using a Zeiss 510NLO upright confocal microscope.

### Image analysis and statistics

Images were analyzed offline using IQ software (Andor, UK). The mean MCB-GSH fluorescence intensity was quantified by selecting regions of interest (ROI size 8.4 μm^2^) over the different cell types in the organ of Corti in single image planes XY. Care was taken to ensure that ROIs were placed within the boundaries of individual cells and for each image file (from an individual N experiment) the following cells were sampled: 3–5 inner hair cells, 12–15 outer hair cells, 4–6 pillar cells, and 4–6 inner sulcus cells. The mean background intensity was measured from regions devoid of cells from within the same image stacks using 4 different ROIs. All mean intensity values from the ROIs were exported to Excel. The background values were subtracted from the measured MCB-GSH values within each data set. The images were not manipulated in any way and measurements were made from single planes. Ratios were calculated from the absolute measurements from individual cells after background subtraction. Unless indicated data are presented as the mean ± SEM and the statistical tests used are either Student's paired *t*-tests, with significance set at *p* < 0.05 or ANOVA with *post-hoc* Tukey-Kramer's, with significance set at *p* < 0.05.

## Results

### Cellular GSH expression in the organ of corti during cochlear maturation and aging in the mouse

**Figure 1:**
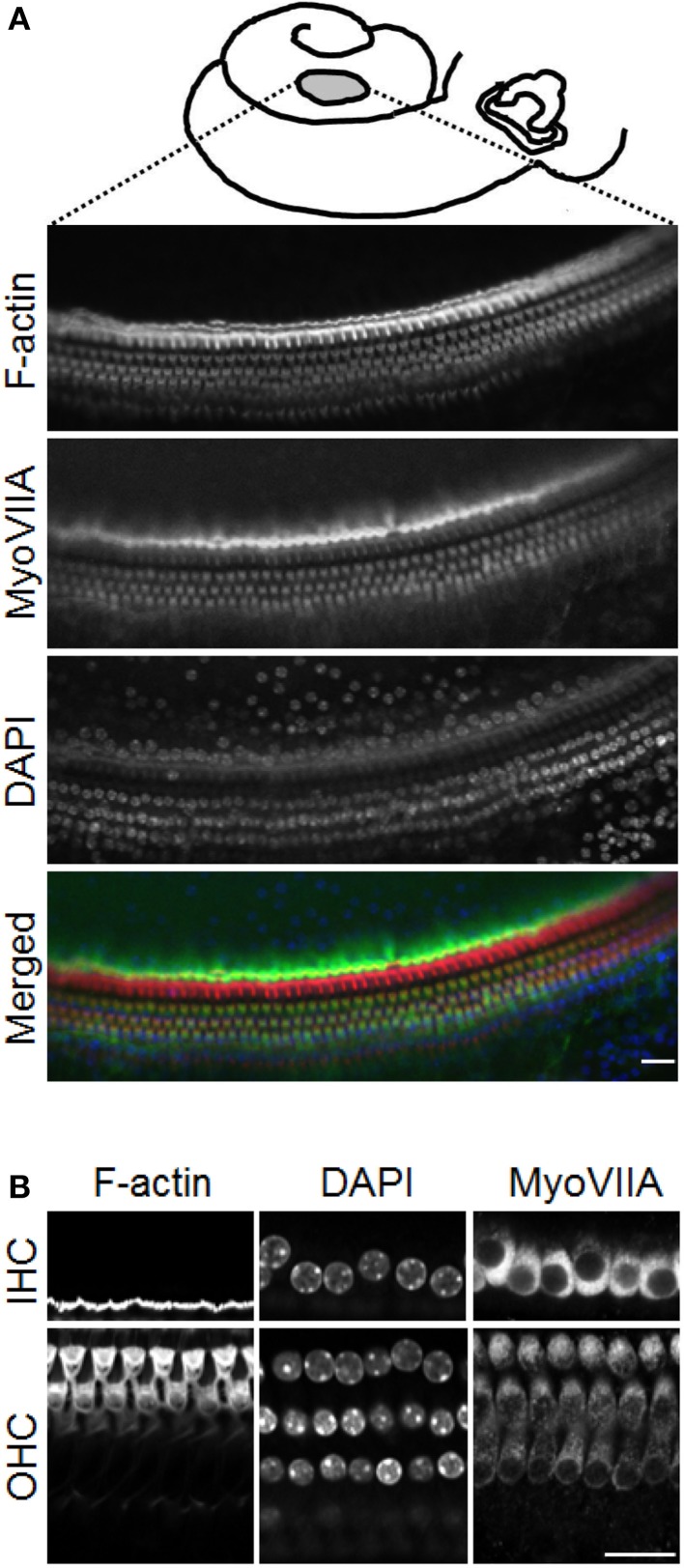
**Hair cell status in the *ex vivo* auditory bulla preparation. (A)** Schematic diagram showing the position of the opening made in the mid to apical turn of the mouse cochlea. Lower planes, confocal optical slices showing inner and outer hair cells labeled with phalloidin (for F-actin, red), DAPI (nuclei, blue) and immunolabeled for myosin VIIA (MyoVIIA, green), and a color merge of the different channels. **(B)** Higher magnification images of inner (IHC) and outer hair cells (OHC). Scale bars: **(A)** 20 μm, **(B)** 20 μm.

**Figure 2:**
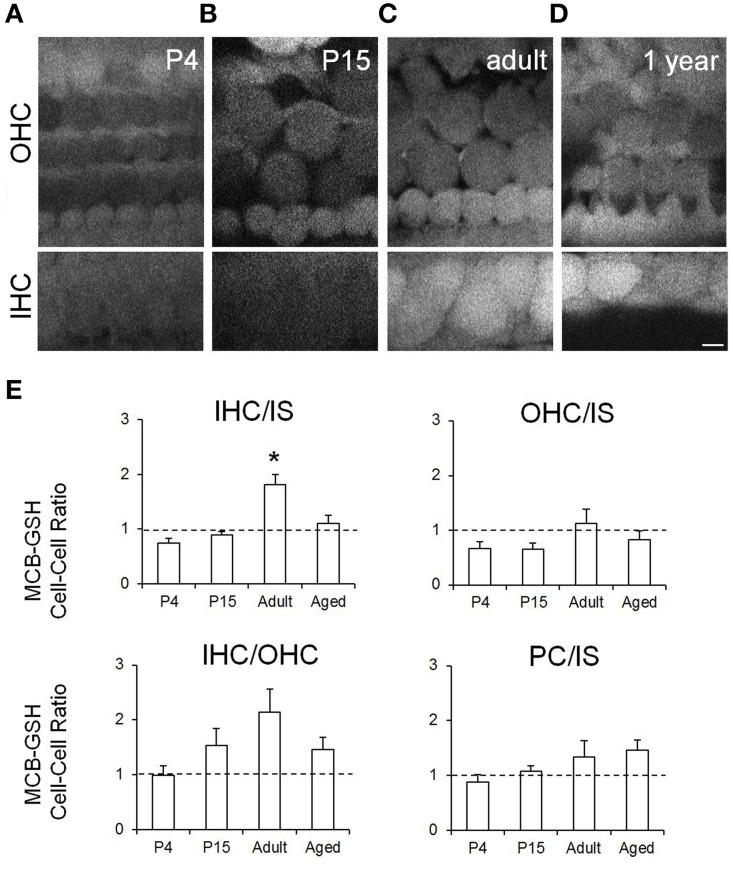
**Live imaging and quantification of MCB-GSH staining in the organ of Corti in auditory bulla explant preparations from mice at different stages of hearing development**. Higher fluorescence intensities indicate greater cellular GSH content. Representative multiphoton single image planes from **(A)** P4 mice; **(B)** P15 mice; **(C)** adult mice (P30), and **(D)** ~1 year old (aged) mice. Scale bar: 5 μm. **(E)** MCB-GSH fluorescence quantification in OHCs, IHCs, inner sulcus cells (IS) and pillar cells (PC). Ratio values were quantified for hair cells and the IS cells (IHC/IS, OHC/IS), between the two hair cell types (IHC/OHC) and between two supporting cell types (PC/IS). Data are presented as mean ± SEM; *n* = 4 (P4–P6); *n* = 5 (P14–P16); *n* = 6 (P33–P41); *n* = 5 (P357–P424) ANOVA Tukey-Kramer's test.; ^*^p < 0.05.

To evaluate the GSH content of cochlear cells at different ages we used an acute whole auditory bulla preparation from mice in combination with live-imaging. In this preparation a window is made in the cochlear bone, providing optical access to the sensory epithelium, the organ of Corti, *in situ*, at all ages. The preparation maintains the native architecture of the tissue and important cell-cell interactions and causes minimal damage to the organ of Corti (Figure 1). Acutely explanted bullae were incubated with monochlorobimane (MCB) which forms a fluorescent adduct with reduced GSH. At steady state this fluorescence is proportional to the cellular GSH content. Live images of MCB-GSH fluorescence were acquired using multiphoton imaging in bullae from mice of different ages ranging from an immature stage prior to hearing onset (P4–6), at hearing onset (P14–16), in young adults (P33–47) and in ~1 year old mice (Figures 2A–D). The GSH distribution varied across the different cell types in the organ of Corti with the most obvious differences being between supporting cells and hair cells, consistent with our recent work in neonatal cochlear explants (Blacker et al., 2014). With increasing age, we observed an increase in GSH content in hair cells, with the inner hair cells showing the most obvious increase relative to surrounding cells. Quantification of GSH-MCB fluorescence across a number of different bullae preparations at each age revealed a trend for increased GSH content in outer and inner hair cells with maturation followed by a reduction after 1 year, but we note that these changes did not reach significance. GSH levels in the inner sulcus cells were quite stable across the four ages tested, with mean absolute levels of 194 ± 55, 102 ± 63, 155 ± 72, and 129 ± 88 at P4–6, P14–P16, adult and 1 year old respectively. A potential source of variation comes from loading and optical access in the bullae thus by calculating the GSH ratio between cells within the same bullae the inter-preparation variability in loading is reduced. We first calculated intensity ratios between the hair cells and the inner sulcus supporting cells in the same preparations for all the different ages. The GSH content ratio between the inner hair cell and inner sulcus cell increased from more than two fold between the P15 animals and the adult animals (Figure 2E). We also calculated ratios between the inner and outer hair cells and in adult animals we found that inner hair cells had approximately twice the level of GSH compared to outer hair cells. Finally we calculated the ratio between pillar cells (another supporting cell type) and the inner sulcus cells and found no significant changes across ages.

### Cellular GSH content in organ of corti explant cultures

**Figure 3:**
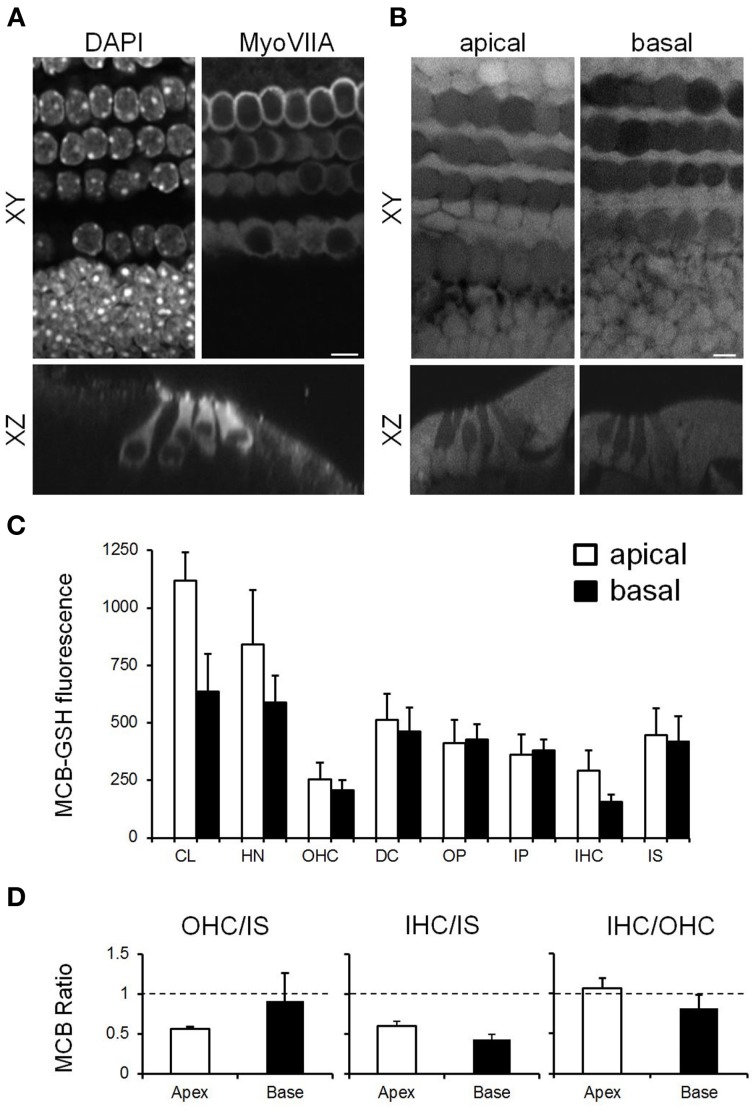
**Characterization of GSH content in cochlear explant cultures from P4–P6 mice (A) Confocal images from cultures fixed and immunolabeled with MyoVIIA and DAPI, showing MyoVIIA-positive inner (lower row) and outer hair cells (upper three rows) in XY and XZ profiles. (B)** Multiphoton images from MCB loaded live cochlear cultures showing the relative GSH content in the apical and basal cochlear coil coils. Scale bar: 5 μm. **(C)** Quantification of GSH content in the apical and basal cochlear turns. Absolute values measure in apical (white bars) vs. basal (black bars) turn cochlear cells: CL, HN, OHCs, DC, OP, IP, IHCs, and IS cells. **(D)** Average ratio values between cells within data sets for OHCs/IS, IHCs/IS, and IHC/OHC. Data presented are mean ± SEM; *n* = 3–6 different cochlear explants from different animals for apical and basal cells.

Longer term experiments are not possible in the adult auditory bulla preparation since the hair cells do not survive over longer periods and also we were limited to accessing the upper middle to apical turns of the cochlea. Therefore, in order to assess the effects of ototoxins such as aminoglycosides we switched to using explant cultures from P4–P6 mice. The explant cultures maintain the cellular arrangement of the organ of Corti such that they are comparable with that seen in the acute bullae at that age (see Figures 1A, 3A; Figures 2A, 3B). We assessed the GSH content of cochlear cells in this experimental preparation by incubating the immature explants with MCB using the same methodology as described for the bullae preparation. There were no significant differences in GSH expression between cell types when measured in the explant cultures compared to acute bullae preparations from the same age mice (Figures 2A, 3B). In the explant cultures we were able to image almost the whole length of the cochlea. There are known differences in susceptibility to ototoxins and to noise damage between the basal (high frequency) and apical (low frequency) ends of the cochlea. Therefore, we compared GSH content in cells from the basal and apical turns (Figure 3B). A complete analysis of GSH content in all of the cell types in the of organ of Corti: Claudius cells (CL), Hensen's cells (HN), outer hair cells (OHC), DC, outer pillars (OP), inner pillars (IP), inner hair cells (IHC), and inner sulcus supporting cells (IS) revealed that although cells exhibit different absolute MCB-GSH fluorescence intensity values there were no significant differences in GSH content in cells of the same type from the apical vs. basal turns (Figure 3C). To compare GSH levels between cells we also obtained data by ratioing values within data sets, as we did for the *ex vivo* bulla preparations. This analysis also failed to show any significant differences between apical and basal coil cells (Figure 3D). During these experiments we did observe GSH content within the actin-rich hair cell stereocilia in both the explant cultures and in the acute bullae preparations (Supplementary Figure 2).

### The role of GSH in neomycin toxicity

**Figure 4:**
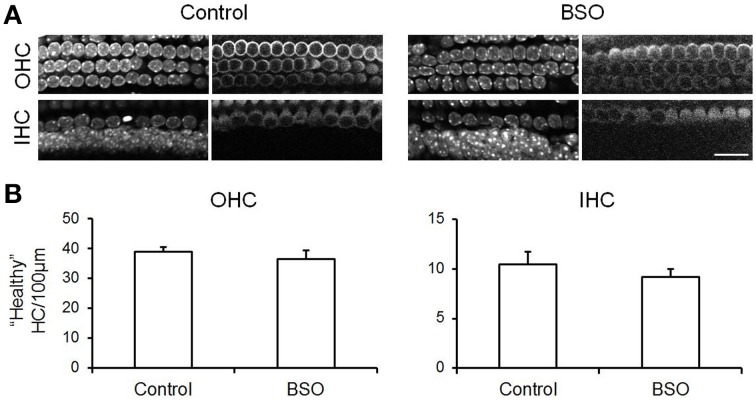
**Depletion of cellular GSH using BSO does not cause hair cell death. (A)** Confocal images of the OHC and IHC regions in control and BSO-treated cochlear cultures immunolabeled with MyoVIIA (right panels) and stained with DAPI (left panels). **(B)** Quantification of “healthy” hair cells in control and BSO-treated samples per 100 μm length of the cochlea. Cells were considered to be healthy if they exhibited normal chromatin and were positive for MyoVIIA. Data are presented as mean ± SEM. N for control (8) and BSO-treated (9). Student's *t*-test, revealed no statistical differences. Scale bar: 20 μm.

**Figure 5:**
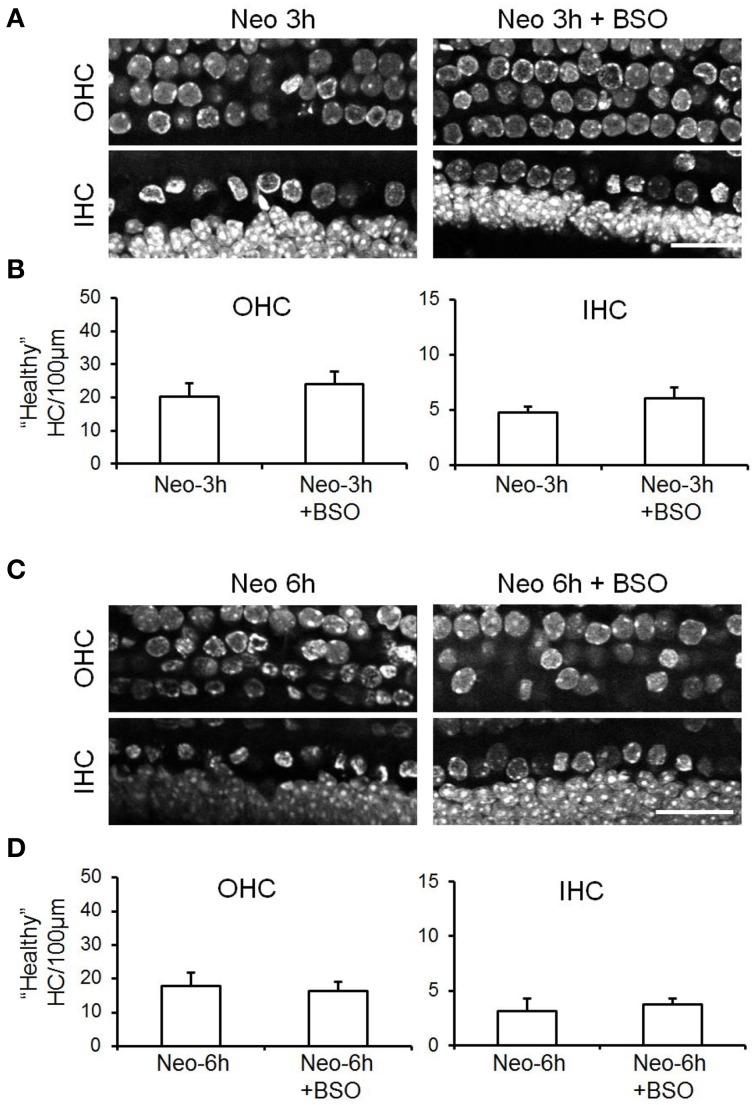
**Depletion of cellular GSH does not enhance neomycin induced-hair cell death. (A)** Examples of confocal images of DAPI-stained OHC and IHC in cochlear explant cultures treated with neomycin for 3 h compared with those that had been pre-incubated with BSO for 18 h to deplete GSH before neomycin treatment. **(B)** Quantification of “healthy” hair cells per 100 μm length in control or BSO-treated cultures exposed to neomycin for 3 h. **(C)** As above for **(A)** except that neomycin cultures were fixed after 6 h exposure to neomycin. **(D)** Quantification of “healthy” hair cells per 100 μm length in control or BSO-treated cultures exposed to neomycin for 6 h. Data are presented as mean ± SEM. N for Neo-3h (7), Neo-3h+BSO (7), Neo-6h (6) Neo-6h+BSO (8). Student's *t*-test's revealed no significant differences. Scale bar: 20 μm.

Hair cells do not survive in the *ex vivo* whole bullae preparations from adult mice for longer than ~12 h. However, the hair cells in cochlear explant cultures from immature mice are viable for many days allowing us to address whether reducing cellular GSH levels enhanced neomycin-induced hair cell toxicity. Cellular GSH concentration was reduced by incubating cochlear explants in buthionine sulfoximine (BSO) for 18 h. After the BSO treatment, GSH content was evaluated in each cell type in cochlear explant cultures. GSH was reduced by between 65 and 85% across the different cell types with inner hair cells showing the least reduction and outer hair cells the greatest (Supplementary Figure 3). Untreated control and BSO-treated explants were immunostained with a hair cell antibody (myosin VIIA) along with markers for chromatin (DAPI) and for F-actin (phalloidin). Counts of healthy hair cells were made on the basis of positive myosin VIIA staining combined with a healthy nucleus and these revealed that BSO treatment alone for 18 h does not result in hair cell death over this time period (Figure 4). In subsequent experiments, explants (control or BSO-treated) were then subjected to neomycin-treatment for 3 or 6 h (Figure 5). There was a ~50% reduction in the number of healthy hair cells from control levels (10 inner hair cells and 39 outer hair cells per 100 μm length of the cochlea) at both 3 and 6 h, with the latter showing the greatest reduction (compare Figures 4, 5). However, we did not observe any significant difference in the numbers of healthy hair cells after neomycin treatment between explants in which GSH had or had not been depleted by BSO-treatment (Figure 5).

## Discussion

In this study, we evaluated cellular GSH distribution in organ of Corti cells at different ages, from P5 to ~1 year old animals, using an *ex vivo* whole auditory bulla preparation. A window was opened in the apical region to allow optical access to the cells and for the MCB loading that was used to assess GSH content. Using live multiphoton imaging we determined cellular GSH concentrations in individual cells, overcoming some limitations of biochemical and histological approaches to measuring GSH in intact cells and tissue (Sun et al., 2006). For example, previous studies showed that tissue fixation, when using antibodies to measure GSH, lead to potential alterations in GSH status (Hjelle et al., 1994). Here, using our loading conditions the MCB reaction was at steady state, and thus our measurements of the fluorescent MCB-GSH product reflect cellular GSH levels. We note that we have not attempted to determine GSH levels in subcellular compartments in the present experiments.

The *ex vivo* whole auditory bulla preparation has been used previously both in guinea pigs (Mammano and Ashmore, 1993) and in mice (Tiede et al., 2007, 2009). It is a useful approach to study the organ of Corti in a native but *ex vivo* context. Here we first characterized the hair cell status in this preparation using the expression of the hair cell marker myosin VIIA and assessed the stereocilial bundles using phalloidin. These same assays were applied after MCB assessment of cellular GSH content to confirm the normal cellular status of all the preparations used in this study.

GSH is one of the most important cellular antioxidants. GSH is distributed in mitochondrial, cytosolic and ER pools where it maintains exogenous antioxidants such as vitamins C and E, peroxide and glutaredoxins in their reduced and active forms. Alterations in cellular GSH have been linked to variety of neurodegenerative diseases such as Parkinson's, Alzheimer's, Huntington's, amyotrophic lateral sclerosis and others (Schulz et al., 2000; Wilson and Shaw, 2007). Moreover, a low level of GSH results in cellular vulnerability toward oxidative stress products, especially in neurons (Murphy et al., 1990; Bolaños et al., 1995; Dringen et al., 1999).

GSH is the major free radical scavenger in the brain, and it has found at higher levels in glial cells compared to the neurons (Keelan et al., 2001). Here we observed a similar difference in the relative GSH content between supporting cells and hair cells in the immature organ of Corti. This finding is consistent with our previous data from the neonatal cochlea which also indicated a higher concentration of enzyme-bound NADPH in pillar cells compared to OHCs (Blacker et al., 2014). It is important to note that NADPH is the co-factor involved to restore GSSG to GSH (Ying, 2008). A potential analogy between supporting cells and glial cells has been proposed by a number of studies (Lahne and Gale, 2008; Monzack and Cunningham, 2013; Mellado Lagarde et al., 2014) and, for the most part, the data herein provide additional support for this suggestion. Whether supporting cells might provide GSH for the hair cells, as has been proposed for glial cells and neurons, remains to be determined. However, here we show that there is an increase in inner hair cell GSH content from the immature stage to when the hearing system is fully functional in the young adult animal. Thus, in the adult, the ratio of cellular GSH levels in inner hair cells compared to supporting cell is reversed. Interestingly, the GSH content of outer hair cells did not increase significantly during this maturation period, although we note that the ratio of inner to outer hair cell GSH content also did not change significantly, indicating perhaps, that the outer hair cell GSH content does increase with age but that the change is more discrete than observed in inner hair cells. By 1 year of age, the GSH content in inner hair cells relative to the supporting cells is reduced. The C57BL/6 mice used in the present study exhibit early onset hearing loss and this may influence these results. However, our measurements were made at the apical end of the cochlea (<10 kHz frequency region) where both the hair cell and the hearing loss is relatively minimal at 12 months (Spongr et al., 1997). A reduction in GSH content with aging has been indicated in HPLC studies on auditory nerves from 3 and 24 months old rats, where it was reduced by as much as 86% (Lautermann et al., 1997). In addition and by association, mitochondrial SOD2 protein expression is reduced by 50% and the levels of glutathione-conjugated proteins (potential indicators of oxidative stress) increased three fold in cochleae from aged mice compared to young animals (Jiang et al., 2007).

GSH content in hair cells isolated from the guinea pig cochlea was measured using MCB (Sha et al., 2001). In that study it was noted that longer outer hair cells, presumed to be from more apical turns, had a greater GSH content than shorter outer hair cells from the basal coils. Unfortunately no direct comparison with inner hair cells was made although it was noted that inner hair cells were considered to be healthier after the isolation procedure, consistent with a higher GSH level and a resistance to cellular stress. The latter observation could also simply have been due to the dissociation procedure being less traumatic for inner hair cells compared to outer hair cells. Previous measurements of GSH in cochlear explants from neonatal rats indicated that there are cell to cell differences (Blacker et al., 2014). Here we show measurements made in the adult cochlea that reveal higher expression of cellular GSH in inner hair cells compared to outer hair cells and also that this enhanced GSH expression appears to develop during cochlear maturation. Higher GSH levels compared to outer hair cells could explain the comparative resistance of inner hair cells to ototoxic agents and to noise trauma.

GSH levels have been strongly implicated in providing a protective role in the cochlea and our data showing raised cellular GSH in inner hair cells correlates with this assertion. Food deprivation results in a reduction in endogenous GSH and has been correlated with enhanced auditory dysfunction (Hoffman et al., 1987) and supplementation with glutathione monoethyl ester has been shown to ameliorate noise-induced hearing loss in animals on a low protein diet (Ohinata et al., 2000). GSH also slowed the progression of gentamicin ototoxicity when applied as a co-therapy along with the aminoglycoside *in vivo* (Garetz et al., 1994). Further support for an important role for GSH in protecting the cochlea from trauma or toxicity comes from GSH peroxidase (Gpx1) knock out mice that exhibit loss of hair cells and also spiral ganglion neurons in the basal cochlear turn. When those mice were subjected to noise trauma they exhibited increased hearing loss compared to wild-type controls (Ohlemiller et al., 2000). Thus, part of the data presented herein along with a number of other papers have implicated GSH in providing protection from various types of auditory stress. A reduction of ROS generation within hair cells themselves has been suggested although rarely tested directly.

Here we evaluated the role and potential importance of cellular GSH during aminoglycoside ototoxicity. We performed these longer term experiments in cochlear explant cultures from postnatal day 4 to 6 mice. Explants were pre-treated with BSO for 18 h in order to deplete GSH and depletion was confirmed by MCB labeling. After 18 h of BSO-treatment explants were then challenged with 1 mM neomycin for 3 or 6 h. Compared to controls not treated with BSO, there was no significance difference in hair cell survival or death with either 3 or 6 h of neomycin exposure. If anything, we identified a trend for hair cell survival in the BSO-treated explants at the earlier 3 h time point, which was not present after 6 h treatment. The simplest explanation for these data is that ROS production is not as major a determinant of aminoglycoside-induced hair cell death as has been suggested. A number of reports have begun to indicate that mechanisms other than ROS generation may be critical in promoting cell death during ototoxicity. Recent work has indicated that aminoglycosides may block cytoplasmic ribosomal activity (Francis et al., 2013) rather than targeting mitochondria, and this would be consistent with ROS playing a less significant role in hair cell death. Moreover, recent experiments undertaken in live zebrafish have shown that the endoplasmic reticulum (ER) is the primary target of aminoglycosides rather than mitochondria and that ER to mitochondrial transfer of Ca^2+^ may well be responsible for catastrophic mitochondrial depolarization (Esterberg et al., 2013, 2014). Such mitochondrial depolarizations have, in the past, been suggested to result from ROS generation but those data argue strongly against that. Whether ROS contribute to the effects on the ER that have been described in zebrafish hair cells remains to be determined.

An alternative explanation for the lack of effect of GSH depletion on neomycin-induced hair cell death describe here, is that when cellular GSH is reduced, other free radical scavenging systems take over so that that GSH depletion does not cause any additional hair cell death. Although this is possible, we predict that we should still have observed some effect of the GSH depletion in these experiments. A further investigation into potential regulation of other cellular antioxidants present in sensory hair cells is warranted.

In summary, using live imaging to measure cellular GSH levels we observe increased cellular GSH in inner hair cells compared to outer hair cells, but also higher levels in supporting cells compared to the outer hair cells. These data are consistent with the enhanced sensitivity of outer hair cells compared to inner hair cells with various types of cellular stress, from noise, ototoxic drugs to aging. However, when we tested the role that cellular GSH plays during neomycin-induced hair cell death we were unable to provide positive data in support of GSH-dependent protection. Further work is required to determine whether other ROS scavenging systems are able to provide sufficient buffering to cover for the lack of GSH in our experimental condition or whether we need to consider that ROS play a lesser role in aminoglycoside-induced hair cell death than has been previously suggested.

### Conflict of interest statement

The authors declare that the research was conducted in the absence of any commercial or financial relationships that could be construed as a potential conflict of interest.

## Abbreviations

GSH: reduced glutathione, glutathione, glutamylcysteinylglycine
GSSG: glutathione disulphide
MCB: monochlorobimane
ROS: reactive oxygen species
BSO: l-buthionime sulfoximine
CL: Claudius cells
HN: Hensen's cells
OHC: outer hair cells
DC: Deiters' cells
IHC: inner hair cells
HC: hair cells
OP: outer pillar cells
IP: inner pillars cell
IS: inner sulcus.
