# Supplementary material for: Cellular glutathione content in the organ of Corti and its role during ototoxicity
Source: Front Cell Neurosci. 2015 Apr 28;9:143. doi: 10.3389/fncel.2015.00143 (PMC4412067; doi:10.3389/fncel.2015.00143)
Supplement: Supplementary file 1 [file Image1.PDF]

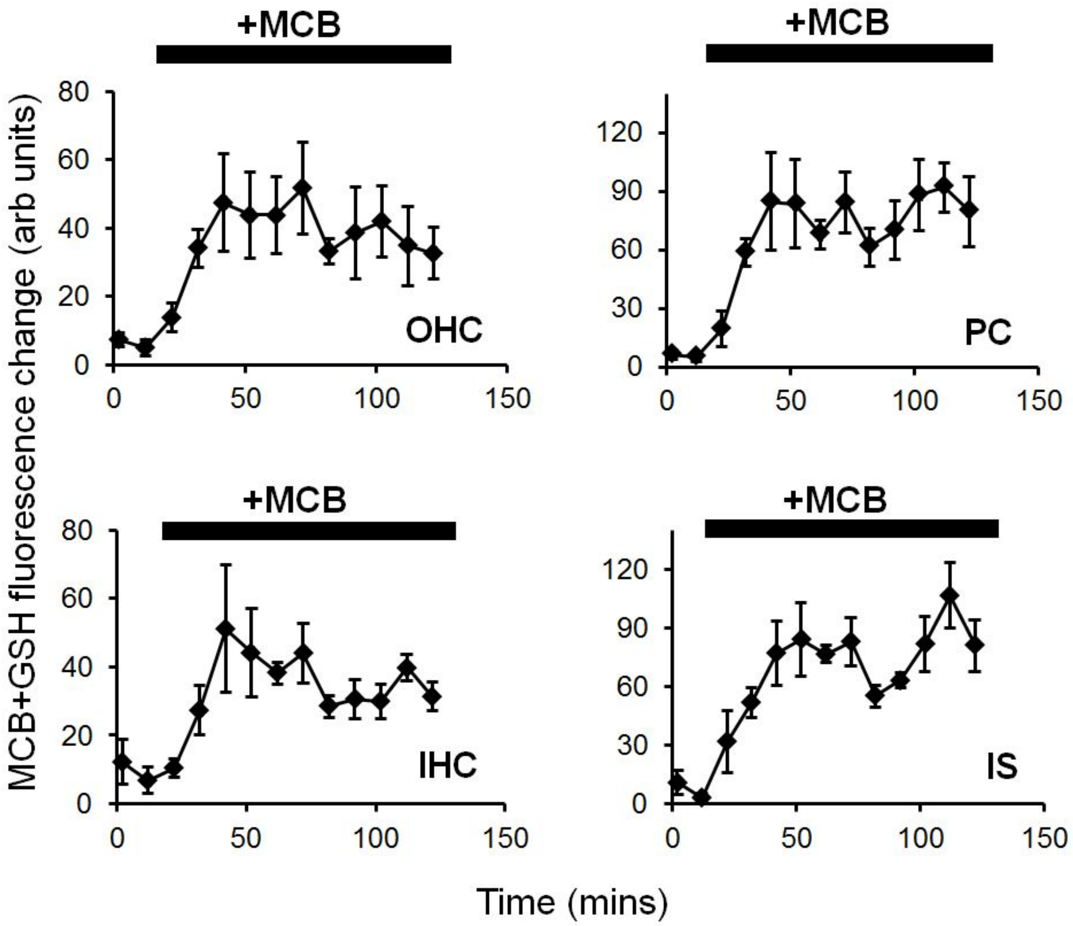

Supplementary Figure 1: MCB loading kinetics in the organ of Corti. MCB-GSH fluorescence image stacks were acquired were acquired every 10 minutes for 120 minutes. Twenty minutes after the onset of the recording 50 µM MCB was applied. The four graphs show the change in MCB-GSH fluorescence measured in four different cell types, outer hair cells (OHC), pillar cells (PC), inner hair cells (IHC) and inner sulcus cells (IS). Data represent mean ± SEM from 4 different explants.
