# Supplementary material for: Cellular glutathione content in the organ of Corti and its role during ototoxicity
Source: Front Cell Neurosci. 2015 Apr 28;9:143. doi: 10.3389/fncel.2015.00143 (PMC4412067; doi:10.3389/fncel.2015.00143)
Supplement: Supplementary file 2 [file Image2.PDF]

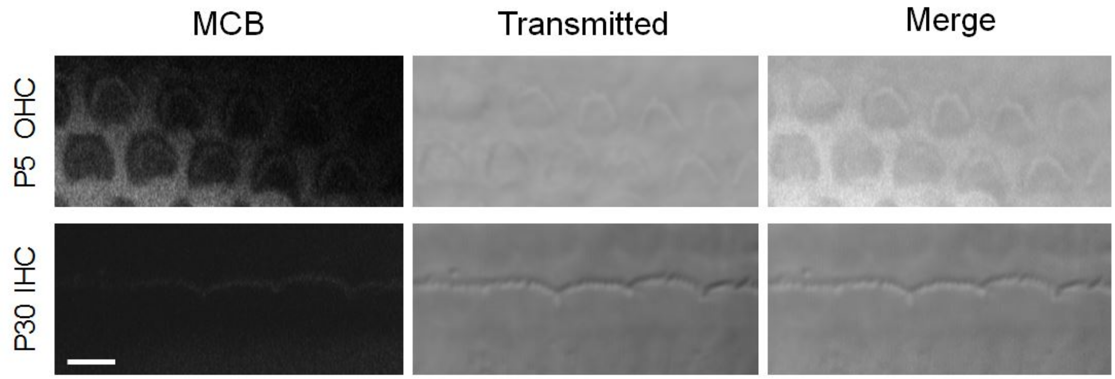

Supplementary Figure 2: GSH expression in hair cell stereocilia. Single plane multiphoton images of MCB-GSH fluorescence combined with transmitted light images. Upper panels show the apical surfaces of outer hair cells from a P5 cochlea. Lower panels show the stereocilia in inner hair cells from a P30 cochlea in the auditory bulla preparation Scale bar, 5 µm.
