# Supplementary material for: Cellular glutathione content in the organ of Corti and its role during ototoxicity
Source: Front Cell Neurosci. 2015 Apr 28;9:143. doi: 10.3389/fncel.2015.00143 (PMC4412067; doi:10.3389/fncel.2015.00143)
Supplement: Supplementary file 3 [file Image3.PDF]

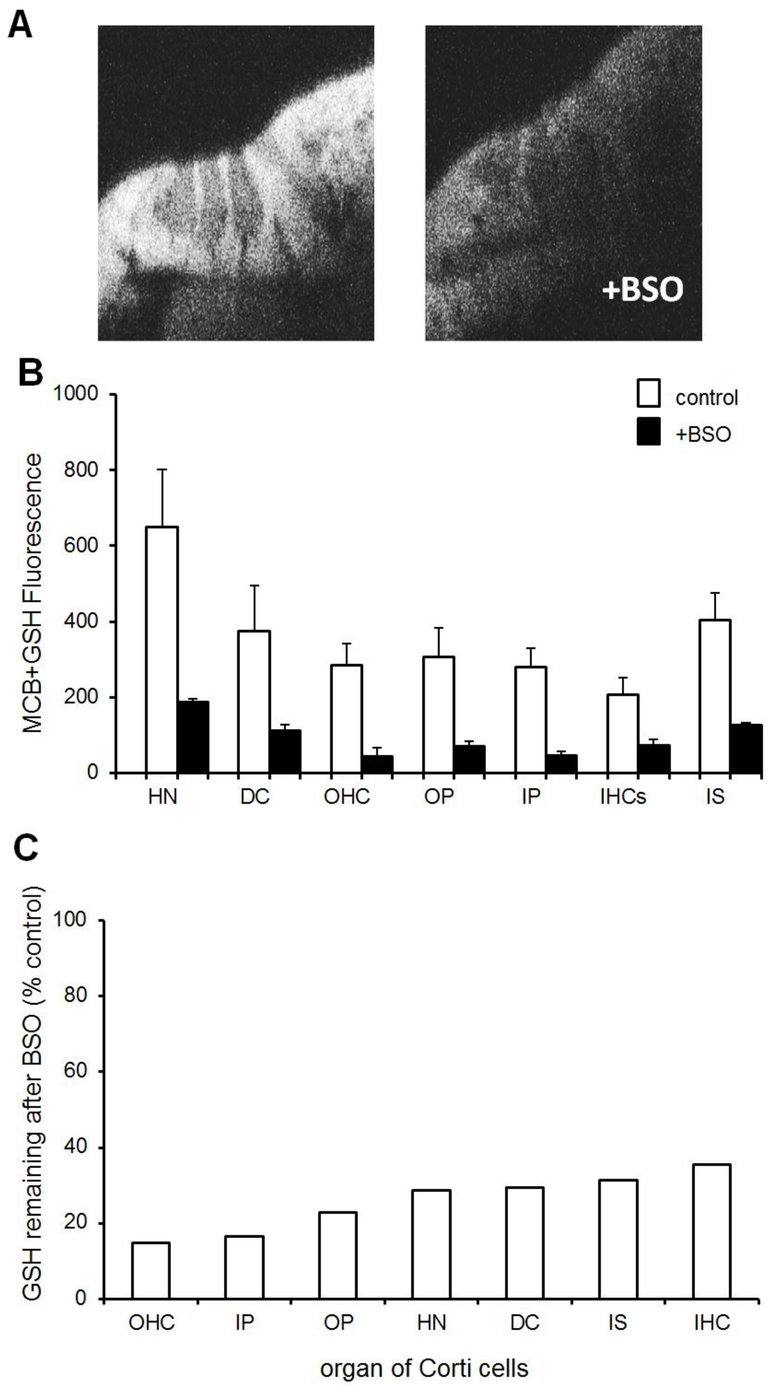


**Supplementary figure 3:** GSH depletion after BSO treatment. (A) Multiphoton XZ images of MCB-GSH fluorescence in control and BSO-treated cochlear cultures. (B) Quantification of cellular GSH content in various types of cochlear cell from a number of untreated control and BSO-treated preparations. Hensens cells (HN) outer hair cells (OHC), Deiters, cells (DC), outer pillar cells (OP), inner pillar cells (IP) inner hair cells (IHC) and inner sulcus cells (IS). (C) Ranked % control cellular GSH levels after reduction by BSO treatment in the different cell types. Data are presented as mean ± SEM. N numbers: control (11) and BSO treated (4). The change elicited by BSO is significant ($p < 0.05$, Student's (one tailed) t test) for all cell types.
